# CO2 solubility and composition data of food products stored in data warehouse structured by an ontology

**DOI:** 10.1016/j.dib.2023.108950

**Published:** 2023-02-05

**Authors:** Melanie Munch, Patrice Buche, Luc Menut, Julien Cufi, Valérie Guillard

**Affiliations:** a I2M, U. Bordeaux, INRAE, Talence, France; b UMR IATE, University of Montpellier - INRA, 2 place Pierre Viala F-34060 Montpellier Cedex, France; c LIRMM, Université de Montpellier, CNRS, INRIA GraphIK, Montpellier, F-34060, France

**Keywords:** CO2 Solubility, Ontology, Food, Compositional parameters, Knowledge graph

## Abstract

This data paper presents the values of CO_2_ solubility at different temperatures and main compositional parameters (protein, fat, moisture, sugars and salt content) for food products from different categories: dairy products, fishes and meats. It is the result of an extensive meta-analysis gathering the results of different major papers published on the domain on the period of 1980 to 2021, presenting the composition of 81 different food products corresponding to 362 solubility measures. For each food product, the compositional parameters were either extracted directly from the original source, or extracted from open-source databases. This dataset has also been enriched with measurements made on pure water and oil for comparison purposes. In order to ease the comparison between different sources, data have been semantized and structured by an ontology enriched with domain vocabulary. They are stored in a public repository and can be retrieved through the @Web tool, a user-friendly interface allowing to capitalize and query the data.

**Specifications Table**

**Table:** 

Subject	Biochemistry

Specific subject area	Food science and food engineering
Type of data	Table
How the data were acquired	Solubility data and their temperature of measurement were acquired through an extensive monitoring on the Web of Science (https://www.webofscience.com/) by three reviewers using the keywords “CO_2_ solubility” and “Food” conducted twice a year over 2016 to 2021, which constituted a database of 21 references from 1980 to 2021. We focused on three types of products: cheese, meat and fish. For all measures, compositional data was added from the articles if available. In case of missing (or, in some cases, incomplete) information, the dataset was complemented using the MultiDB explorer tool (https://ico.iate.inra.fr/meatylab), that compiles several diverse open-sourced databases. Using the food product's appellation, we manually selected, in the MultiDB explorer tool [27], the food product that seemed the closer to the one mentioned in the original article in order to retrieve the corresponding compositional parameters (water, fat, proteins, sugar, salt) to fill in the database. The dataset was also enriched with CO_2_ solubility measurements and their temperature on pure water and oil.
Data format	Analysed
Description of data collection	Data are structured by the @Web Ontology, which allows to describe relations between concepts. We have 362 CO_2_ solubilities, temperatures and nutritional composition for 81 food products from 21 publications. All values (solubilities and compositional values) are linked to their sources: in case of missing value in the publication, the name of the open-sourced database used to complete data is indicated.
Data source location	INRAE, PLANET-IATE, 1208. Agropolymers and Emerging Technologies Facility, Montpellier FR-34,060, France https://doi.org/10.15454/1.5572338990609338E12
Data accessibility	Data are accessible in a public repository Repository name: INRAE dataverse (https://data.inrae.fr/) Data identification number: 10.15454/4SFE64 Direct URL to data: https://doi.org/10.15454/4SFE64
Related research article	M. Münch, V. Guillard, S. Gaucel, S. Destercke, J. Thévenot, P. Buche, Composition-based statistical model for predicting CO_2_ solubility in modified atmosphere packaging application, J. of Food Engineering. 340 (2023). https://doi.org/10.1016/j.jfoodeng.2022.111283

## Value of the Data


 

• Collection of a significant and up-to-date complete dataset including the CO2 solubility, as well as the measurement temperature and the food product's composition. When missing, the composition was retrieved from the Multi-DB tool, which helped us to find composition of similar food products by compiling several open-accessed databases.
• In order to ease the comparison between the different format, all data have been semantized and structured by a single ontology, which enrich it by adding new queryable metadata.
• All data from this dataset can be traced to their original source, guaranteeing a trackability of the different information, in accordance of the FAIR data principle.
• Due to its bacteriostatic effect, CO2 is a key component for modified atmosphere packaging. However, the experimental determination of its solubility is long and costly. This dataset offers an exhaustive collection of state-of-the-art results which can greatly benefit ongoing research.
• All the stakeholders of the food engineering field can benefit from this dataset for modelling gas transfer in food. These data could also serve as benchmark for other researchers dealing with characterization and modelling of CO2 transfer in food.
• In addition of the food engineering aspect, this dataset is also of interest for other knowledge bases that can be enriched with Linked Open Data.

## 1 Objective

This dataset aims to improve a previously published dataset [2] composed of 72 measures of CO_2_ solubility by adding new information (the food products composition) as well as 290 new measures taken from the literature. This original dataset presented CO_2_ measures made only on cheese products. In this new dataset, we define a complete and more exhaustive base (by extending the range of presented food products) from which models could be designed in order to link a food product's composition to its CO_2_ solubility [1]. Moreover, the work done on the semantization of its data (using the @Web ontology) defined a knowledge base that can be further augmented, either by adding new measures or linking it to other knowledge bases. Each data can also be easily associated to its source, which allows an easy comparison of the results. Thus, the main objective of this dataset was to provide a way of browsing complex and heterogenous data from multiple sources on the subject of food products’ CO_2_ solubility. Due to the complex nature of its evaluation, CO_2_ solubility's measure is costly and time consuming. This article aims to help ongoing research by providing an easy-to-access and to-browse dataset in order to help developing new evaluation method to predict this value for different kinds of food products. This represents a major breakthrough for modified atmosphere packaging studies, and thus food products’ conservation and preservation. From a long-term perspective, this dataset can help evaluate new kinds of packaging as replacement of the plastic current ones, and thus help design new, more environmentally friendly packaging.

## 2 Data Description

This dataset is the updated version of a previous dataset [2]. It introduces 362 CO_2_ solubilities measured over 81 different food products (distributed over the cheese, meat, and fish categories, plus water and oil). For each solubility, its corresponding measurement temperature was also kept, since according to the corresponding literature it has a strong impact on the measured value [3].

**Fig. 1:**
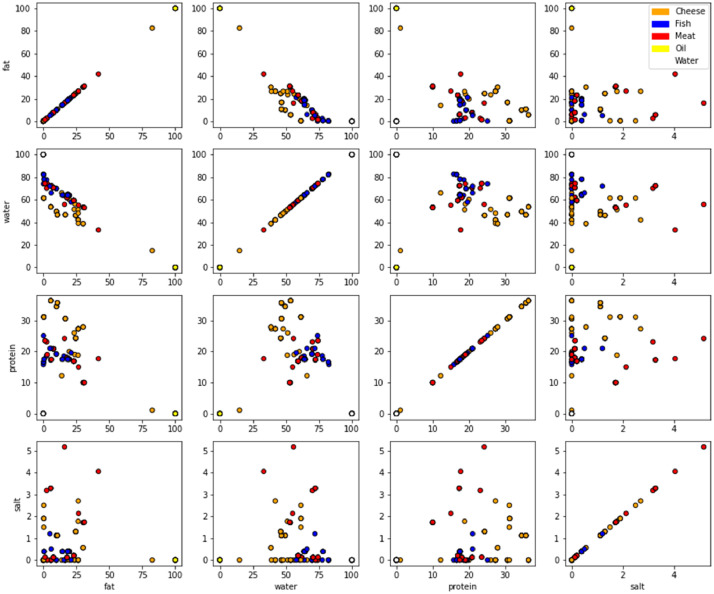
Detail of the 81 different food products composition.

Each food product is described by its nutritional composition: the five most important constituents (water, fat, proteins, salt and sugars) are kept. Fig. 1 presents an overview of the diversity of our products’ compositions. It is important to note that not all combinations of these compositional parameters are represented due to the physical-chemical limit of food products: for instance, products with a salt quantity over 5% are rather scarce.

Data are stored as a knowledge graph structured by the @Web Matter Transfer ontology, which is dedicated to represent n-ary relations (i.e., relations to link multiple concepts together) in the field of mass transfer, especially gas (e.g. O_2_ and CO_2_), in food and packaging [4], [5], [6]. In our case, two relations are defined: a first to link a food product to its solubility to CO_2_, and a second to link the same food product to its nutritional composition. Moreover, values are structured such that they are always associated with a unit, which allows comparison between the different entries.

Finally, @Web guarantees trackability of the sources by describing groups of products as documents, identified by their bibliographical reference. In the end, a food product is always semantically linked to its properties and original source.

The knowledge graph can be accessed manually through the @Web interface (https://data.inrae.fr/dataverse/atweb); it can also be directly queried using the SPARQL language in the accessible SPARQL endpoint (https://ico.iate.inra.fr/fuseki/annotation/query).

However, for the sake of simplicity, we also provide in this article a csv file referencing our main contributions: for each solubility, we indicate its unit in the original article, the temperature of measurement, the food product considered and its composition (as well as the source if the composition is not from the original paper). We also indicate the article's title, authors and date of publication, and finally its @Web identifier. This csv file is supported by the Python script used to build it, which details relevant SPARQL queries.

Table 1 presents an overview of the amount of data considered and how it is distributed between the references.

**Table 1:** Overview of the references and the amount of data.

Reference	Data Type	Amount	Reference	Data Type	Amount
[7]	CO_2_ Solubility	21	[8]	CO_2_ Solubility	6
	Composition	5		Composition	1
[9]	CO_2_ Solubility	20	[10]	CO_2_ Solubility	9
	Composition	5		Composition	1
[11]	CO_2_ Solubility	5	[12]	CO_2_ Solubility	3
	Composition	1		Composition	3
[3]	CO_2_ Solubility	3	[13]	CO_2_ Solubility	18
	Composition	3		Composition	18
[14]	CO_2_ Solubility	1	[15]	CO_2_ Solubility	24
	Composition	1		Composition	8
[16]	CO_2_ Solubility	6	[17]	CO_2_ Solubility	4
	Composition	6		Composition	1
[18]	CO_2_ Solubility	22	[19]	CO_2_ Solubility	46
	Composition	3		Composition	6
[20]	CO_2_ Solubility	1	[21]	CO_2_ Solubility	5
	Composition	1		Composition	1
[22]	CO_2_ Solubility	2	[23]	CO_2_ Solubility	5
	Composition	1		Composition	1
[24]	CO_2_ Solubility	13	[25]	CO_2_ Solubility	137
	Composition	7		Composition	4
[26]	CO_2_ Solubility	11			
	Composition	4			

## 3 Experimental Design, Materials and Methods

We first considered measures of CO_2_ solubility from the original article.

To extend this first dataset, a corpus on the subject of food products CO_2_ solubility has been defined, using the PRISMA guidelines (http://www.prisma-statement.org/). This corpus is the result of an extensive monitoring by three reviewers on the Web of Science (https://www.webofscience.com/) using the keywords “CO_2_ solubility” and “Food”, conducted twice a year over 2016 to 2021, to constitute a database of 21 references from 1980 to 2021.

From this corpus, CO_2_ solubilities were extracted as well as their temperature of measurement (all units were kept from the original source), and added to the original dataset.

In a second time, we manually complemented each food product with their composition in principal food nutrients (fat, water, proteins, salt, sugar):

- If directly available in the article, it was retrieved as such.
- If missing, the MultiDB Explorer tool [27] was used to complete the composition.

**Fig. 2:**
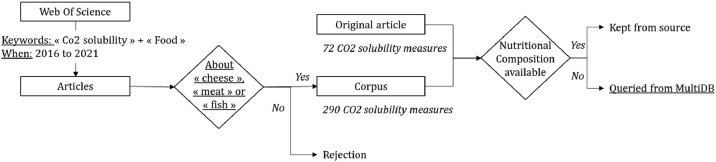
Process of selection for our dataset. Underlined terms highlights times where decisions are dependant of subjective choices made by the reviewers.

MultiDB explorer tool comprises a database gathering nutritional information of various food products from seven different available sources, such as the Composition of Foods Raw, Processed, Prepared USDA National Nutrient Database for Standard Reference (Release 27) or the ANSES-CIQUAL French food composition table (2017). Using the food product denomination in the article, we queried missing nutritional information. In this case, the chosen product's identifier used in MultiDB explorer tool is indicated in our dataset for a better trackability, as well as the original database. This process of selection is illustrated with Fig. 2.

To be noted, in some cases the nutritional composition was partially indicated in the original article. In this case, the known information was kept, and used to guide the search of the closest food product.

## Ethics Statements

This work neither involves human subject nor animal experiments.

## CRediT authorship contribution statement

**Melanie Munch:** Data curation, Writing – review & editing, Visualization. **Patrice Buche:** Conceptualization, Methodology, Software, Data curation, Writing – review & editing. **Luc Menut:** Software, Data curation. **Julien Cufi:** Software. **Valérie Guillard:** Funding acquisition, Conceptualization, Investigation, Writing – review & editing.

## Declaration of Competing Interest

The authors declare that they have no known competing financial interests or personal relationships that could have appeared to influence the work reported in this paper.

## Data Availability

CO2 solubility and composition data of food products annotated from the scientific litterature (Original data) (https://entrepot.recherche.data.gouv.fr/)
